# Exploring Ginseng Bioactive Compound’s Role in Hypertension Remedy: An In Silico Approach

**DOI:** 10.3390/ph18050648

**Published:** 2025-04-28

**Authors:** Sagar Kurmi, Rita Majhi, Hilal Tayara, Kil To Chong

**Affiliations:** 1 Department of Electronics and Information Engineering, Jeonbuk National University, Jeonju-si 54896, Jeollabuk-do, Republic of Korea; kurmisagar@jbnu.ac.kr (S.K.); majhirita@jbnu.ac.kr (R.M.); 2 School of International Engineering and Science, Jeonbuk National University, Jeonju-si 54896, Jeollabuk-do, Republic of Korea; 3 Advanced Electronics and Information Research Center, Jeonbuk National University, Jeonju-si 54896, Jeollabuk-do, Republic of Korea

**Keywords:** ginseng plants, phytochemicals, hypertension, molecular docking, molecular dynamics simulation, network pharmacology

## Abstract

**Background/Objectives**: Ginseng has been a traditional remedy for centuries, known for its diverse benefits such as anti-inflammation, antioxidant, bactericidal, fungicidal antidiabetic, and anticancer effects. This study employs a network pharmacology approach with molecular dynamics simulation to investigate the potential mechanisms through which ginseng-derived compounds control hypertension. **Methods**: The total of 70 bioactive compounds were identified from the literature and classified as ginsenosides, which fall under Protopanaxadiol-type ginsenosides, Protopanaxatriol-type ginsenosides, and Ocotillol-type saponins. The target proteins related to hypertension were collected from the drug bank, and interactions between proteins network were examined using STRING 12.0 and Cytoscape 3.10.1. Bioinformatics tools were used to analyze the biological enrichment of genes. The core targets extracted through network pharmacology were subjected to molecular docking studies. Similarly, the docking score below −6.0 kcal/mol was further visualized by performing molecular dynamics simulation to see the binding affinity between the complexes. Finally, pharmacokinetics and toxicity of the compounds were evaluated using computational tools. **Results**: Molecular docking and simulation results revealed that Floralquinquenoside C, Ginsenoside Rg6, Notoginsenoside T1, and Floralquinquenoside B exhibited strong binding and stability with Angiotensin-converting enzyme (ACE) and Carbonic Anhydrase-I (CA-I), which alters the renin–angiotensin system, calcium signaling pathway, adrenergic signaling in cardiomyocytes, c-GMP-PKG signaling pathway, etc., to regulate high blood pressure. **Conclusions**: The results show that the phytochemicals from ginseng could act as potential candidates for the management of hypertension, which may help minimize the side effects caused by synthetic anti-hypertensive drugs available on the market.

## 1. Introduction

High blood pressure (HBP) also known as hypertension is a clinical state characterized by elevated blood pressure levels within the walls of arteries [1]. It is an alarming cardiovascular threat that comprises heart attack, ischemia, vision loss, renal diseases, and stroke. Annually, around 9.4 million deaths are caused worldwide due to hypertension and related consequences [2]. There are numerous synthetic anti-hypertensive drugs available on the market such as angiotensin-converting enzyme (ACE) restrain, diuretics drugs, antagonist for calcium, and angiotensin receptor. However, these synthetic drugs can cause several adverse effects, including dry cough, constipation, diarrhea, and fatigue [3]. Lifestyle plays an important role in maintaining hypertension. Factors such as smoking, alcohol consumption, being overweight, lack of physical exercise, and high salt intake are the main contributors to increased blood pressure [4,5]. Many scientists have employed natural compounds for treating diverse ailments due to their pharmacological and therapeutic health advantages in mitigating such effects [6]. Therefore, several studies have suggested natural plant-based drugs such as *Panax ginseng*, *Crocus sativus*, *Andrographis paniculata*, *Nigella sativa* etc. can help lower blood pressure by minimizing side effects [7,8].

**Figure 1:**
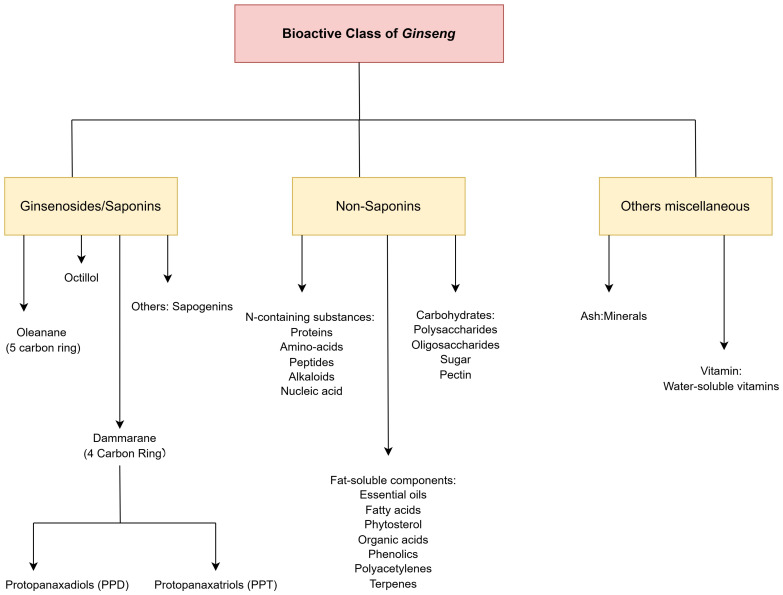
Overall phytochemicals class present in ginseng.

The species like *Panax quinquefolius* L., *Panax notoginseng* (Burk.) F.H. Chen, and *Panax ginseng* C.A. Meyer which belongs to family Araliaceae have been used to cure and treat various diseases due to their pharmacological and clinical health benefits [9,10]. These ginseng species have been used as traditional medicines for centuries in East Asia, especially in countries like Korea, Japan, and China. Traditionally, ginseng has been used to boost immunity and energy, reduce fatigue and stress, and also to cure a variety of ailments. *P. ginseng* and *P. quinquefolius* are commonly used as tonics and stimulants to promote life. Similarly, *P. notoginseng* is used for blood clotting and also for injury recovery. Additionally, the flowers of these species are often consumed as foods or drugs to treat digestive disorders, metabolic imbalance, and respiratory conditions [10]. Ginseng plants are rich in phytochemicals, especially ginsenosides, a class of dammarane-type triterpenoid saponins defined as protopanaxatriol-type ginsenosides (PPTs). These compounds are responsible for various therapeutic remedies, especially in cardiovascular applications. Furthermore, other phytochemicals present in these species of ginseng, such as phenolics, flavonoids, vitamins, anthocyanin, etc., contribute to their anti-inflammatory, antioxidant, antibacterial, antiviral, and antifungal activities as shown in Figure 1 [10,11,12]. These properties make ginseng plants some of the most valuable and widely used plants in therapeutic systems. Ginseng plants have many pharmacological benefits, such as stress and neurological disorders (Alzheimer’s, Parkinson’s, hypertension, chronic heart failure, and Huntington’s disease) [13,14]. The therapeutic efficacy of ginseng has been extensively studied in recent decades, especially when it comes to curing a number of diseases such as heart disease, high blood sugar condition, sexual and cognitive dysfunction, respiratory disease, immunological disorders, and neurological disorders [12,15]. The current state of drug discovery is driven by its potential for accelerating the therapeutic breakthrough and minimizing the need for laboratory tests.

Several promising methods and tools are now used to predict the effectiveness of compound compositions against a wide range of diseases, minimizing the cost and time associated with extensive laboratory experiments [16]. One such emerging field is network pharmacology, which is often used in conjunction with molecular docking to study the interaction of drugs and targets and stimulate the process of therapeutic innovation in a more efficient way [14,17]. Techniques like molecular docking and simulation are often applied to investigate potential new compounds, which helps to analyze interactions such as hydrogen bonds, hydrophobic bonds, pi-pi stacking, etc. [18].

The aim of this study is to introduce an in silico framework for assessing the anti-hypertensive effects of bioactive compounds derived from multiple *Panax* species rather than single species. This approach offers a broader pharmacological insight and reflects the potential combined effects of bioactive compounds. As a result, this study provides a system-level perspective that differs from previous studies, which have typically focused on a individual molecule or pathway. Furthermore, this study investigates the relatively unexplored CA-I target for hypertensive treatment while also elaborating on the significance of the hypertensive control by the Renin–Angiotensin System. This approach extends beyond bridging traditional herbal therapy and contemporary pharmacology by providing an in silico platform for hypothesis-driven experimental validations of findings, which builds off existing work on anti-hypertensive pharmaceutical drugs derived from natural sources. Thus, the objective of this study is to evaluate the anti-hypertensive activity of ginseng compounds through the use of an in silico method. First, the compounds are screened and tested for drug likeness (DL ≥ 0.18) to find the most promising candidates. Ultimately, the protein targets are predicted with Swiss Target Prediction and genes related to hypertension are obtained from the Drug Bank. In addition, some overlapping targets are found and enrichment analyses are performed to find the functional relevance such as KEGG pathway analysis and Gene Ontology (GO) analysis. After constructing a protein–protein interaction (PPI) network, hub genes are detected through centrality measures including degree, betweenness, and closeness. Besides docking, molecular dynamics simulations are carried out on the complexes with docking scores under threshold −6.0 kcal/mol to assess complex stability. To verify that the screened top compounds are safe and effective, pharmacokinetic parameters and toxicity evaluations are conducted on the best screening compounds.

## 2. Results

### 2.1. Collection of Phytochemicals, Target Proteins, and Genes

Based on data obtained from the literature and the Drug Bank database, a total of 70 compounds presented in Table S1 and 32 and target proteins presented in Table S2 were initially collected. To ensure the drug-likeness properties, the compounds were filtered using a threshold value of DL ≥ 0.18, resulting in retention of 54 compounds. Additionally, a total of 644 target proteins were collected for the study. Among these, 612 target proteins were predicted to be associated with ginseng-derived bioactive phytochemicals. Simultaneously, 32 hypertension-related targets were extracted from the Drug Bank.

### 2.2. Intersection Gene Analysis

**Figure 2:**
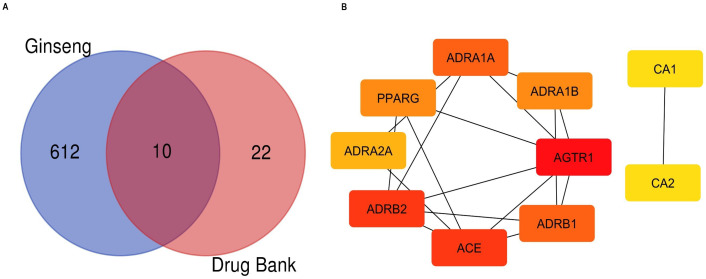
Network pharmacology analysis. (**A**) Venn diagram showing the overlap between compound targets and disease targets. The top 10 intersection represent potential therapeutic targets, indicating possible significance in treatment; (**B**) Top ten targets ranked by their network centrality. Node color indicates their relative importance, with red color indicating higher centrality and yellow indicating lower centrality.

The common target genes between hypertension and ginseng-derived compounds were identified using Venn diagram analysis. A total of ten overlapping genes were identified, as shown in Figure 2A. The protein–protein interaction (PPI) network was constructed using the STRING database and found to contain 10 significant nodes that were linked by 17 interaction edges. The resulting PPI network is shown in Figure 2B. The top ten genes were retrieved based on topological parameters, as shown in Table 1. It presents the top 10 genes ranked by their degree, betweenness centrality, and closeness centrality in a network analysis. Betweenness centrality is the way to measure how often a node (gene/protein) lies on the shortest path between two other nodes. A node with a high betweenness value is an important hub that connects different parts of the network and mediates important signaling. In contrast, nodes with low betweenness contribute minimally to network connectivity and are often located on the periphery. Genes such as *AGTR1*, *ADRB2*, *ACE*, and *ADRA1A* exhibited high betweenness values, suggesting that they may serve as key mediators within the network and could be promising drug target candidates due to their role in inter-node communication. Similarly, closeness centrality measures how close a node is to all other nodes in the network based on the shortest paths. High value closeness is the node that can quickly interact with or influence all other nodes. It is usually well-connected and central. As shown in Table 1, *CA1* and *CA2* have maximum closeness centrality value (1.000), suggesting that they are centrally located within the network and capable of quickly transmitting signals across it. Conversely, nodes with lower closeness centrality are more distant from others, resulting in slower communication [19]. These top ten genes represent key target genes that are commonly associated with both ginseng bioactive compounds and hypertension, and they may serve as important therapeutic targets.

### 2.3. GO and KEGG Enrichment Pathway Examination

**Figure 3:**
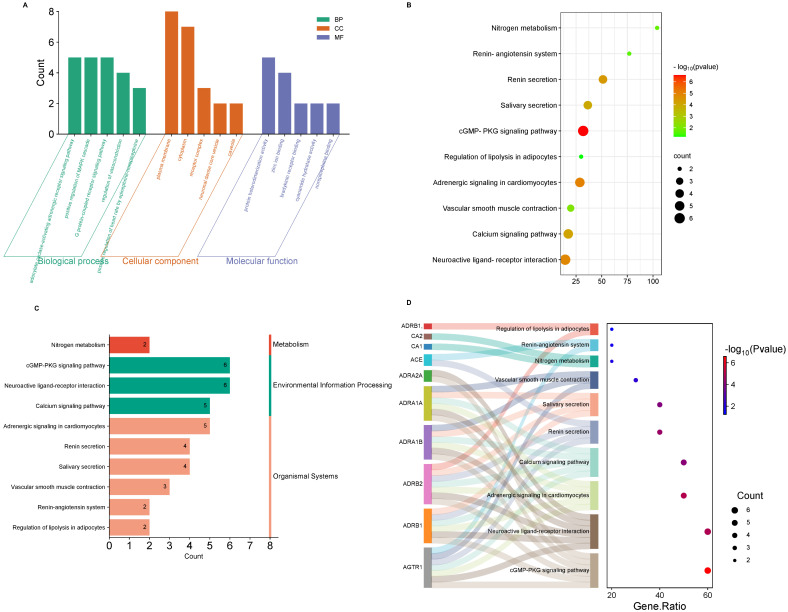
Enrichment analysis and pathway mapping of ginseng target genes. (**A**) Gene Ontology (GO) classification of the 10 overlapping target genes based on three categories: Biological Processes (BPs), Cellular Components (CCs), and Molecular Functions (MFs); (**B**) KEGG pathway enrichment bubble plot showing the top pathways associated with the target genes. The x-axis represents gene ratio (number of genes enriched in a pathway relative to total genes). (**C**) A bar chart categorizing enriched KEGG pathways by function: Metabolism, Environment Information Processing, and Organismal Systems; (**D**) Enrichment significance and gene pathway association. Sankey plots on the left show mapping between target genes and their associated pathways. Pathway enrichment analysis is shown on the right bubble plot, with bubble size representing gene association and color gradient representing *p*-value, with red showing the most significant pathways.

**Figure 4:**
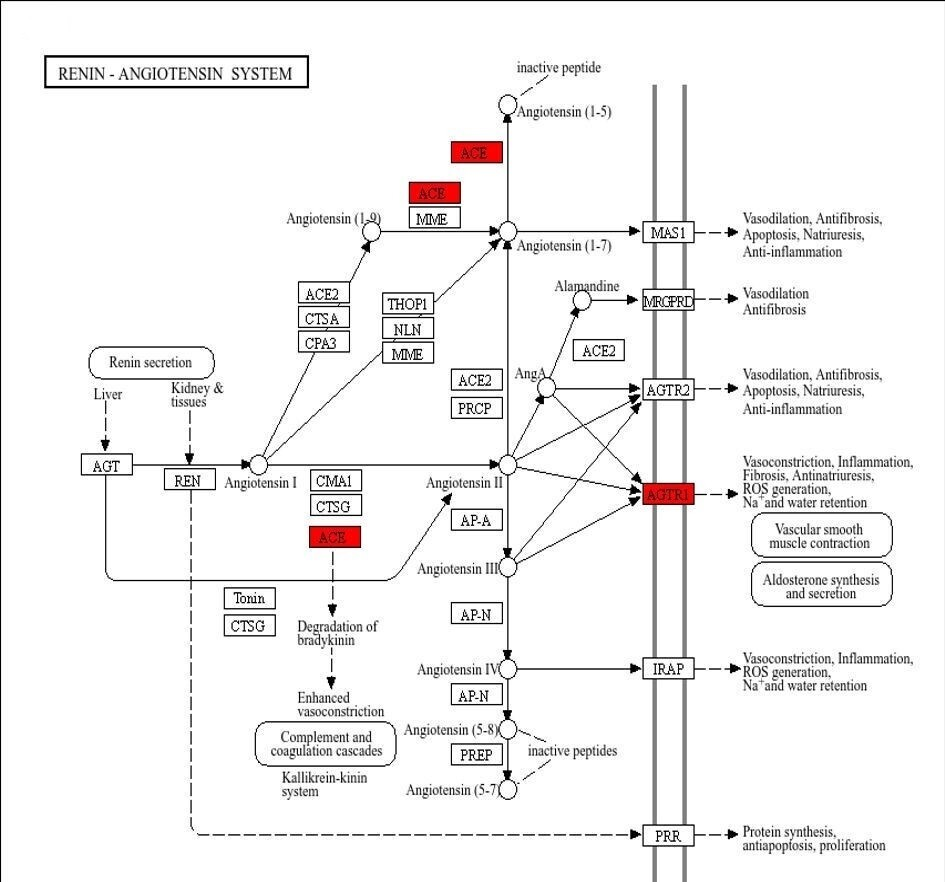
Renin–angiotensin pathway (RAS). The diagram shows key steps in the RAS pathway involved in blood pressure and fluid regulation. The red highlighted targets (*ACE*, *AGTR1*) are key genes identified in this study making them potential therapeutic targets.

Gene enrichment analysis revealed 34 biological processes, 9 cellular components, and 8 molecular functions. The top five enrichment of GO terms are listed in Table S3. Probability value < 0.05 was used to obtain a biological process [20] indicating statistically significant enrichment with gene representation exceeding 20%. Ginseng compounds and high blood pressure targets are associated with different biological process, cellular components and molecular function as shown in Figure 3A. The Kyoto Encyclopedia of Genes and Genomes (KEGG) pathway analysis further highlighted key pathways, including the calcium signaling pathway, adrenergic signaling in cardiomyocytes, and the renin–angiotensin system, as shown in Figure 3B. Moreover, Figure 3B shows enriched biological pathways based on statistical significance and gene count. The red-colored pathways are the most significantly enriched (lowest *p*-values), indicating strong involvement in the biological process studied. The green-colored pathways are less significant. Larger dots represent pathways involving more genes. This analysis highlights key pathways that may play crucial roles in the regulation of blood pressure. Table S4 displays *p*-values, false discovery rates (FDR), and gene ratio across various KEGG pathways. Genes such as *ACE*, *AGTR1*, *ADRB1*, *ADRB2*, and *CACNB2* are the main proteins for the regulation of blood pressure and are involved in pathways such as the renin–angiotensin pathway, the adrenergic pathway, and the calcium signaling pathway. Figure 3C,D highlights the distribution of genes across metabolic, signaling, and systemic pathways. The significance of these results was supported by gene counts and corresponding *p*-values, which confirmed the involvement of several hub genes in major blood pressure regulatory mechanisms. Figure 4 provides a detailed representation of the renin pathway, with the key hypertension-associated genes marked in red.

### 2.4. Molecular Docking Study

**Figure 5:**
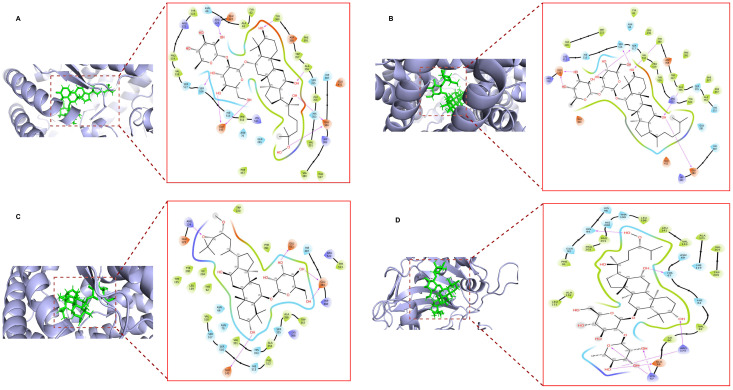
Molecular interaction diagram between ligand and protein. (**A**) Floralquinquenoside C within the binding pocket of the ACE receptor; (**B**) Ginsenoside Rg6 within the binding pocket of the ACE receptor; (**C**) Notoginsenoside T1 within the binding pocket of the ACE receptor; (**D**) Floralquinquenoside B within the binding pocket of the Carbonic anhydrase-I receptor.

Compounds such as Floralquinquenoside C, Ginsenoside Rg6, Ginsenoside Km, Notoginsenoside T1, Ginsenoside Ki, Floralginsenoside M, and Floralquinquenoside B disclosed promising results with good docking scores ranging from −6.0429 to −7.7578 kcal/mol which are given in Table 2. The binding score from of all the compounds is provided in Table S5. The interactions between ginseng-derived compounds and their corresponding target proteins, including key interacting residues, are illustrated in Figure 5.

### 2.5. Molecular Dynamics Simulation

**Figure 6:**
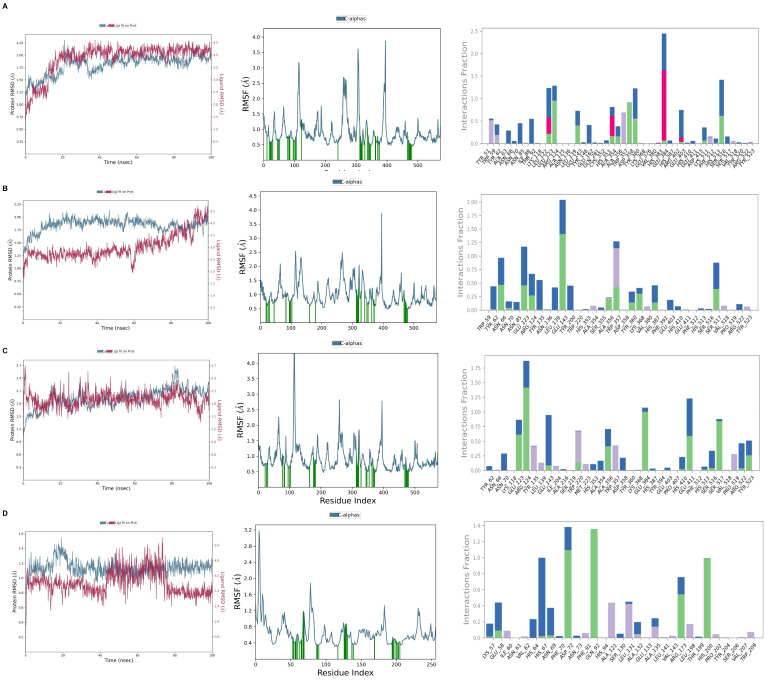
RMSD, RMSF and protein–ligand contact of all ginseng compounds, respectively. (**A**) Floralquinquenoside C; (**B**) Ginsenoside Rg6; (**C**) Notoginsenoside T1; and (**D**) Floralquinquenoside B. Each row presents three plots describing different aspects of the simulation: **Left side:** Root mean square deviation (RMSD) over the simulation time for the protein backbone (blue) and ligand (pink), indicating system stability. **Middle:** Root mean square fluctuation (RMSF) per residue, highlighting the flexibility of amino acid residues across the trajectory. Green bars indicate *α*-helical regions. **Right side:** Protein–ligand interaction fractions throughout the simulation. Interaction types include hydrogen bonds (green), hydrophobic contacts (grey), ionic interactions (pink), and water bridges (blue).

**Figure 7:**
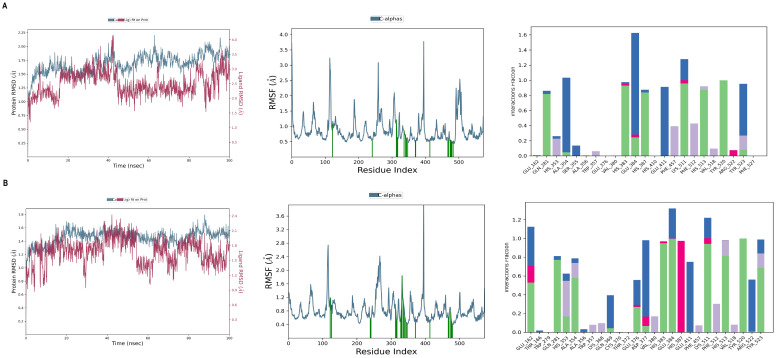
Dynamic simulation of the control drug. (**A**) RMSD, RMSF, and P-L contact of Enalaprilat with ACE protein, respectively; and (**B**) RMSD, RMSF, and P-L contact of Chlorthalidone with Carbonic Anhydrase-I, respectively. These represent the structural deviation, fluctuations, and interaction analyses between control drugs and their target proteins over 100 ns simulation period.

The outcomes obtained after simulation such as RMSD, RMSF, and Protein-ligand contact provide insights into the stability and binding affinity between the complexes. Figure 6 illustrates the detailed molecular interactions of four compounds, including Floralquinquenoside C, Ginsenoside Rg6, Notoginsenoside T1, and Floralquinquenoside B, with ACE and CA-I protein. The lower range of RMSD and lesser amount of fluctuation determine the stable binding of protein and ligand throughout the simulation. Looking at the RMSD graph of selected compounds (Figure 6), the value is below 3 Å, which is in an acceptable range. The lowest RMSD (1.6 Å) was displayed by Floralquinquenoside B with CA-I protein with a stable graph. However, Floralquinquenoside C and the Ginsenoside Rg6 complex have a maximum deviation of up to 2.25 Å and maintain a stable trajectory over the simulation period. In the Notoginsenoside T1 complex (Figure 6C), the highest variation up to 2.7 Å was observed at the beginning and at 85 ns. Between 5 ns and 85 ns, they did not show any significant shift, indicating that they were stable and able to bind with the protein pocket properly. Additional investigation was performed through the RMSF graph (Figure 6). Lower fluctuations with structural integrity were noticed in the RMSF graph. Moreover, Figure 6A,D illustrate lower fluctuation, indicating slight residual movements during contact with the ligand. At last, bonding relations were determined using an interaction fraction diagram. Each type of interaction like hydrogen bonding, hydrophobic bonding, and pi-pi stacking, is crucial for maintaining stability. Interaction fraction values of all compounds exceeded 30%, which means they have good binding with protein. Interestingly, Figure 6A presents diverse, strong interaction forces while other complexes do not have multiple interactions. Residues like GLU 123, ARG 124, TYR 360, GLU 384, and SER 516 were responsible for maintaining stability because their interations ratio is above 1.0. Furthermore, Figure 6B,C diagrams show slightly lower interations fraction compared to Figure 6A. Finally, in the Floralquinquenoside-B complex (Figure 6D), the constant interaction fraction can be seen in residue in HIS 67, ASP 72, GLN92 AND HIS 200. Similarly, to validate the results, docking of the control drugs (enalaprilat and chlorthalidone) and simulation of ACE and Carbonic Anhydrase-I protein were performed. From the RMSD graph between enalaprilat and ACE target, we observed an erratic pattern until 100 ns, but the final RMSD was below 2.25 Å (Figure 7). Looking at the protein–ligand contact, 10 hydrogen bonds were observed. Similarly, in the Chlorthalidone simulation (Figure 7), the RMSD of protein is below 1.35 Å, and initially, the graph followed a stable pattern, but at 80 ns it slightly rose to 1.35 Å and dropped to 1.2 Å. More than four hydrogen bonds were formed during ligand–protein contact for control drugs, which is crucial for the binding affinity of the proteins with the ligands.

### 2.6. *In Silico* ADME and Toxicity Analysis

Table 3 presents the ADME (Absorption, Distribution, Metabolism, and Excretion) analysis of the selected ginseng compounds. Floralquinquenoside C, Ginsenoside Rg6, Floralquinquenoside B exhibited higher water solubility, whereas Notoginsenoside T1 demonstrated moderate solubility. All selected compounds showed low CaCO-2 permeability except Ginsenoside Rg6 (0.569) and Notoginsenoside T1 (0.393). Similarly, human intestine absorption (HIA) was observed to be more than 40% for Notoginsenoside T1 and Ginsenoside Rg6 compared to Floralquinquenoside B (18%), Floralquinquenoside C (19%). Regarding skin permeability, the pkCSM values for all compounds ranged from −2.735 to −2.737, suggesting they possess skin permeability, as ideal values for penetration are typically Log Kp > −2.5. P-glycoprotein is responsible for eliminating toxins and xenobiotics from cells. All compounds were found to be substrates for P-glycoprotein and inhibitors of P-glycoprotein-I. However, none of the compounds inhibited P-glycoprotein-II. From distribution data, all compounds showed a volume distribution value of <−0.15, indicating relatively limited distribution throughout the body. Furthermore, none of the compounds demonstrated the ability to cross the blood–brain barrier (BBB). For CNS permeability, only Floralquinquenoside C and Floralquinquenoside B showed a Log PS > −3, suggesting they can potentially penetrate the central nervous system, whereas the remaining compounds cannot. In the metabolism assessment, pkCSM predicts none of the ginseng compounds inhibit the CYP super-family class. Regarding excretion, all compounds showed a total clearance value > 0.3, indicating efficient elimination from the body. Additionally, none of the compounds were identified as substrates of renal organic cation transporter 2 (OCT2), which is involved in renal drug clearance. Finally, ProTox-3.0 predicted the level of oral toxicity, including hepatotoxicity, carcinogenicity, immunotoxicity, mutagenicity, and cytotoxicity; Table 4.

## 3. Discussion

The prevalence of hypertension remains a significant global health concern and is recognized as a major contributor to heart attacks and mortality worldwide [21]. The most common treatment available on the market is the prescription of synthetic drugs. Studies have demonstrated that angiotensin-converting enzyme (ACE) inhibitors, such as enalaprilat, exhibit strong ACE inhibition in preclinical studies, with in vivo animal studies confirming a potent therapeutic remedy. Chlorthalidone, a thiazide-like diuretic, effectively lowers blood pressure by reducing plasma volume and blood volume, as evidenced in preclinical research. Clinical trials have further validated the efficacy of both drugs, which are now widely prescribed as first-line medications for hypertension by healthcare professionals [22,23]. Enalaprilat in particular is typically administered intravenously due to its poor oral bioavailability [24]. These clinical and preclinical findings provide critical insights into the pharmacodynamics, pharmacokinetics, safety, and mechanisms of action of these anti-hypertensive agents. However, despite their effectiveness, synthetic drugs are often associated with side effects and limited long-term therapeutic benefits in clinical practice [3,25]. This has highlighted the need for the detailed evaluations of drug interactions, contraindications, delivery systems, and optimal dosing strategies. The purpose of this research is to investigates the possibilities of ginseng bioactive compounds as a substitute remedy for hypertension, offering a promising approach to address these limitations (e.g., side effects) by providing alternative treatment options.

Through protein–protein integration analyses, we revealed the top ten possible target genes implicated in the treatment of hypertension, including *AGTR1*, *ADRB2*, *ACE*, *ADRA1A*, *ADRB1*, and so on, as mentioned in Figure 2B. However, among these, only two proteins, ACE and CA-I, emerged as significant targets based on their strong binding scores with the ginseng-derived compounds. The docking score from Table 2 illustrates the good binding interactions of compounds compared to control drugs such as enalaprilat (−6.5 kcal/mol) and chlorthalidone (−7.0 kcal/mol), demonstrating that ginseng-derived compounds bind with target protein [26]. Notably, Floralquinquenoside C and Ginsenoside Rg6 showed the highest binding scores (−7.7578 and −7.5202 kcal/mol, respectively) compared to other compounds. RMSD data illustrate that protein fluctuation is in the acceptable range, meaning protein conformation was more stable throughout the simulation period. Remarkably, the most stable complexes were those formed by Floralquinquenoside C, Ginsenoside Rg6, Floralquinquenoside B, all showing minimal RMSD variation over time. Further assessment of protein flexibility via RMSF and interaction fraction (Figure 6) confirmed this stability. Although some fluctuations peaked up to 4.5 Å, particularly between residue positions 100 and 200 in Figure 6C, overall flexibility was low, indicating preserved structural integrity. Figure 6A,D showed particularly stable regions with low RMSF values. Lastly, interaction fraction analysis revealed that Floralquinquenoside C and Floralquinquenoside B exhibited higher interaction stability compared to Ginsenoside Rg6 and Notoginsenoside T1, with consistent hydrogen bonding and hydrophobic interactions supporting strong ligand–protein binding. In addition, comparative analysis was performed with control drugs such as enalaprilat and chlorthalidone as illustrated in Figure 7. The RMSD value was found to be 2.25 (enalaprilat-ACE complex) and 1.8 Å (chlorthalidone-CA-I complex), with consistent interaction fraction values. These results support the validity of our methodology and highlight the potential of ginseng compounds as viable alternatives to conventional anti-hypertensive drugs. The overall results of the investigation suggest that selected compounds of ginseng are capable of forming stable and strong protein–ligand complexes because of the acceptable RMSD values, consistent interaction profiles, and promising docking scores. However, in order to translate these in silico findings into therapeutic applications, further preclinical and clinical studies are necessary to assess the effectiveness and safety of the drug.

Surprisingly, toxicity assessment results demonstrated that the ginseng phytochemicals were free from toxic effects as presented in Table 4, highlighting their potential safety as a drug supplement. Similarly, the ADME test demonstrates that ginseng compounds such as Ginsenoside Rg6 and Notoginsenoside T1 displayed promising pharmacokinetic properties. However, compounds like Floralquinquenoside C, Floralquinquenoside B exhibited a low intestinal absorption value (<40%), suggesting poor oral bio-availability, as shown in Table 3. Despite this limitation, it was found that all compounds tested were highly water-soluble and were unable to cross the blood–brain barrier (BBB), further increasing the chances of their peripheral safety profile. However, the poor intestinal absorption suggests the need for alternative administered routes required to achieve therapeutic efficacy [27]. On a positive note, all compounds exhibited high total clearance rates, implying efficient elimination from the body and reducing the risk of accumulation and associated toxicity [28]. This emphasizes the significance of in vivo pharmacokinetic research to precisely ascertain how these compounds are absorbed, distributed, metabolized, and excreted in biological system.

Previous studies revealed that ACE is an important enzyme of the renin–angiotensin pathway (RAS), which plays a pivotal role in regulating blood pressure in humans. The hypertension is usually associated with the hyperactivity of the renin, ACE or angiotensin receptor [29]. A primary function of ACE inhibitors is to block the formation of angiotensin II, resulting in increased bradykinin levels that maintain blood pressure and the functional integrity of smooth muscle cells and cardiac myocytes [30]. The findings of this study showed that ginseng-derived compounds may exert anti-hypertensive effects by strongly binding to and inhibiting the ACE enzyme, thereby disrupting the RAS and preventing the formation of angiotensin II. This disruption contributes to the balance of blood pressure, blood volume, and body fluid homeostasis [27,31]. We selected the renin–angiotensin system (RAS) pathway based on both biological relevance to hypertension and significant bioinformatics findings. As shown in Figure 3B, RAS is statistically significant (based on *p*-value and gene count), and multiple genes associated with it. Another point is that RAS plays a crucial role in maintaining sodium water homeostasis and blood regulation, and it is connected with diverse hypertension networks. Figure 3C supports this with a strong gene representation within the organismal systems category. It means they are involved in physiological process like in the cardiovascular and renal system, vascular resistance, and hormonal control. Figure 3D further describes genes (e.g., *ACE*, *CA1*, *CA1AGTR*) involved in the RAS pathway, and these are clinically and pharmacologically targeted genes in hypertension. Thus, this pathway was selected as the central mechanism of interest due to its therapeutic significance in hypertension and consistent support across bioinformatics and simulation data. Drugs available on the market mainly target ACE enzymes to lower BP because angiotensin I is converted into angiotensin II by an enzyme ACE, which is a potent vasoconstrictor that raises blood pressure through vascular constriction, fluid retention, and an increase in sympathetic nervous system activity. This is the process where ACE is involved in the lowering of blood pressure by inhibiting the formation of angiotensin I, leading to vasodilation and aldosterone-mediated fluid retention which ultimately decreases the level of blood volume and leads to lower blood pressure. Among current anti-hypertensive medications, ACE inhibitors, calcium channel blockers, beta-blockers, diuretics, and angiotensin receptor blockers (*ARBs* are the most commonly used. ACE inhibitors remain the front line therapy due to their effectiveness in disrupting the RAS pathway and reducing cardiovascular risk [30,32]. Notably, our results revealed that ginseng compounds such as Floralquinquenoside C, Ginsenoside Rg6, and Notoginsenoside T1 all demonstrated strong inhibitory potential against ACE in molecular docking analyses of the compounds. These results suggest that these compounds may be promising natural ACE inhibitors for the treatment of hypertension.

Moreover, besides ACE, carbonic anhydrase-I has also been identified as an important protein targeted by the ginseng compounds for lowering HBP. It works by catalyzing the transformation of carbon dioxide to bicarbonate. The pH levels of cells, vascular function, and blood flow are regulated by this enzyme [33]. Our results indicate that compound Floralquinquenoside B interacts with carbonic anhydrase-I (CA-I) in blood pressure maintenance. Although *CA1* showed lower degree in the PPI network analysis, docking selection was based on biological relevance to hypertension; in addition, a literature review suggests that *CA1* is involved in vascular function and pH regulation which is an important mechanism for blood pressure control. First, we gathered the target list from the Drug Bank, where CA-I was also included as a target for hypertension management. Additionally, ginseng targets were identified using the Swiss Target Prediction web server, where *CA1* was also listed as a predicted target gene for ginseng compounds. Subsequently, common genes were analyzed using Venn analysis, which yielded a top 10 gene list that included *CA1*. Therefore, we included it in our docking studies. Moreover, the docking scores revealed that the ginseng compound Floralquinquenoside B showed a favorable docking score (−6.5276 k/cal) with the CA-I protein. Furthermore, molecular dynamics simulation results confirmed that Floralquinquenoside B compounds bind to this target protein with good binding affinity. Furthermore, these compounds revealed good pharmacokinetic properties and non-toxicity profiles. Thus, despite its lower centrality in the protein–protein interaction network, *CA1* was incorporated into our docking experiments to evaluate its potential interaction with ginseng-derived molecules from a biological perspective, and the results were promising. These outputs align with the previous research that emphasizes the involvement of protein CA-I in the vascular system and also in maintaining blood pressure [34]. Our data also demonstrated that ginseng compounds and the disease’s main pathway were mainly the RAS pathways. Many studies have also demonstrated that RAS is involved in increasing blood pressure by vasoconstriction and sodium retention [31]. Our finding suggested ginseng compounds might play an important role in controlling a different pathway like RAS, calcium, adrenergic, and c-GMP-PKG as displayed in Figure 3B. In addition, ginseng compounds showed good inhibition for carbonic anhydrase-I protein.

Overall, the findings of our study indicate that ginseng compounds, due to their significant *ACE* and *CA-I* inhibitory potential, favorable safety profiles, and promising pharmacokinetic properties, could serve as viable alternatives to synthetic drugs for hypertension treatment. Furthermore, phytochemicals may help reduce toxicity, enhance tolerability, and lower cardiovascular risks associated with hypertension more effectively than conventional therapies.

## 4. Materials and Methods

### 4.1. Collection of Compound and Target Proteins

The total of 70 ginseng bioactive compounds were obtained from the literature review [35], and 33 target proteins were collected using Drug Bank https://go.drugbank.com/ (accessed 10 May 2024). FDA- approved drugs for hypertension were identified, and the associated target proteins and genes involved in stabilizing high blood pressure were retrieved. After the collection of ginseng compounds, the drug-likeness of each compound, as shown in Table S1, was evaluated using the Molsoft tool https://molsoft.com/mprop/ (accessed 12 May 2024). Similarly, compound-related targets were predicted using Swiss Target Prediction http://www.swisstargetprediction.ch/ (accessed 7 June 2024). Compounds with a drug-likeness (DL) score of ≥0.18 were selected as suitable drug candidates [36].

### 4.2. Determination of Intersection Genes

The disease-related target genes, along with the target genes of ginseng compounds, were entered into the Venn analysis tool found at https://bioinformatics.psb.ugent.be/webtools/Venn/ (accessed 25 June 2024) to visualize the common targets between ginseng and hypertension [37].

### 4.3. Building Protein–Protein Interactions

Our study constructed protein–protein networks which were visualized using the STRING 12.0 database https://string-db.org/ (accessed 15 July 2024) with parameters set to *Homo sapiens* and a minimum interaction score of 0.40. Simultaneously, the centiscape Cytoscape module 3.10.1 https://cytoscape.org/ (accessed 22 July 2024) was applied to calculate the topological parameters of the genes and to visualize the protein network of 32 target proteins. The degree of connection (DC) was used as a key indicator to identify the most significant genes, while the others were classified as secondary indicators [38].

### 4.4. Major Pathway and Gene Function Analysis Through Bioinformatics Tools

We utilized Gene Ontology (GO) analysis to determine the involvement of the top 10 genes in various functional categories, along with their roles and cellular locations. For this evaluation, data were extracted from the DAVID database found at https://david.ncifcrf.gov/tools.jsp (accessed 1 August 2024), and a significant threshold of *p*-value < 0.05 was applied to identify genes significantly involved in different functional levels. In addition, the Kyto Encyclopedias of Genes and Genomes (KEGG) was used to provide information on enriched pathways within the set of genes [20]. Histogram and bar plots were generated using bioinformatic tools using https://www.bioinformatics.com.cn/en (accessed 10 August 2024).

### 4.5. Molecular Docking of Ligands with Their Receptors

First, target proteins were retrieved from the RCSB protein data bank at https://www.rcsb.org/ (accessed 12 May 2024) and compounds were obtained from PubChem, https://pubchem.ncbi.nlm.nih.gov/ (accessed 12 May 2024) database. Using the LigPrep module of Maestro (Schrodinger, 2024), all structures were geometrically refined after being imported into Maestro, and docking commenced with ligand preparation using bioactive compounds derived from ginseng. A total of 54 ligands obtained based on DL ≥ 0.18 as mentioned in Table S6 were processed for docking. To maintain the stability of protein–ligand complexes, we used the OPLS4 force field because it provides accurate binding interaction predictions between molecules [39]. Proteins with PDB ID: 1o86, 3M67, 6I0L, 1I7I, and 6WTH were imported into Maestro. Protein preparation in Schrodinger Maestro involved removing water molecules beyond 5 Å from the co-crystallized ligand, adding missing hydrogen atoms, assigning bond order, and adjusting the protonation states of ionizable residues [40]. Next, a receptor grid was constructed using the prepared protein, centering on a ginseng ligand and setting the grid dimensions to 20 Å from the ligand center [41]. Following the grid generation methodology, the ligands were docked with the corresponding proteins, and binding scores were generated.

### 4.6. Validation of Protein–Ligand Score with Molecular Dynamic Simulation

To ascertain the stability of the protein backbone in the docked complex and to evaluate fluctuations during the contact period along with binding affinity, molecular dynamics (MD) simulations were performed using Maestro (Schrodinger, 2024) [42]. In parallel, the simulation period was set to 100 ns in the Schrodinger Desmond combined with a definite solvent MD package and a fixed OPLS4 force field [40]. An orthorhombic periodic box was used to embed the protein–ligand complex in the solvent, maintaining a minimum distance of 10 Å between the protein atoms and the box boundaries. The TIP3P water model was used to facilitate the solvent application [43]. The system was neutralized with ions such as 0.15 M NaCl, Na+, and Cl−, and the simulation was initiated using NPT ensemble at 300 K and 1.013 bar pressure. Stability was evaluated based on RMSD, RMSF, and bonding interaction outcomes between complexes [44].

### 4.7. *In Silico* ADME and Toxicity Prediction

The ADMET features of four phytochemicals (Floralquinquenoside C, Ginsenoside Rg6, Notoginsenoside T1 and Floralquinquenoside B) were predicted to evaluate their pharmacokinetic responses, which are crucial for minimizing toxicological effects and ensuring favorable drug-like properties. To predict the in silico ADMET properties of these compounds, we used the following tools: pkCSM https://biosig.lab.uq.edu.au/pkcsm/ (accessed 15 December 2024), ProTox-3.0 https://tox.charite.de/protox3/ (accessed 20 December 2024) and ADMETLab 3.0 [45,46].

## 5. Conclusions

Ginseng is a traditional herb that has been used for centuries to treat various diseases due of its valuable bioactive compounds. Our study identifies ginseng compounds as prospective candidates for anti-hypertensive agents by targeting key proteins involved in high blood pressure regulation. A detailed evaluation of the results revealed that ACE and CA-I proteins are potent regulators of the renin–angiotensin system and vascular function, providing a solid basis for blood pressure control. Furthermore, comparison with reference drugs supports our findings and highlights the potential of ginseng compounds as effective alternatives to synthetic medications. Floralquinquenoside C, Ginsenoside Rg6, Notoginsenoside T1, and Floralquinquenoside B showed favorable binding affinities, stable RMSD values, and strong protein–ligand interactions, suggesting probable inhibitory activity against ACE and CA-I proteins. Additionally, ADME analysis showed that the compounds are non-toxic, which is a prerequisite for being safe and therapeutic for treating hypertension. However, some compounds exhibited limitations in terms of poor intestinal absorption, which may require alternative routes of administration to achieve effective therapeutic outcomes. Thus, the current research provides a solid platform for developing ginseng-derived phytochemicals as an effective and safer substitute drug. To fully harness their therapeutic potential, extensive in vitro studies on relevant cell lines and in vivo investigations using animal models are essential to validate the efficacy and safety of these ginseng compounds. Ultimately, well-designed and adequately powered clinical trials in humans are necessary to confirm these promising in silico findings and to thoroughly assess the safety, efficacy, and optimal clinical application of ginseng compounds in the management of hypertension. These future studies will be vital in determining whether ginseng bioactive compounds can indeed serve as a safer and potentially more tolerable substitute or supplement to existing synthetic anti-hypertensive therapies.

## Figures and Tables

**Table 1:** Top 10 genes with degree, betweenness centrality, and closeness centrality.

S. No	Genes	Degree	Betweenness Centrality	Closeness Centrality
1	AGTR1	6	0.158	0.875
2	ADRB2	5	0.071	0.778
3	ACE	5	0.040	0.778
4	ADRA1A	4	0.111	0.700
5	ADRB1	4	0.040	0.700
6	ADRA1B	3	0.016	0.636
7	PPARG	3	0.000	0.636
8	ADRA2A	2	0.016	0.583
9	CA1	1	0.000	1.000
10	CA2	1	0.000	1.000

**Table 2:** Docking scores and hydrogen bonding details for selected compounds.

S. No	Compound Name	CID Number	Docking Score (kcal/mol)	Molecular Weight (g/mol)
1	Floralquinquenoside C	23652173	−7.7578	817.0
2	Ginsenoside Rg6	91895489	−7.5202	767.0
3	Ginsenoside Km	102294900	−6.7204	668.9
4	Notoginsenoside T1	131752527	−6.7279	652.9
5	Ginsenoside Ki	102294899	−6.0429	668.9
6	Floralginsenoside M	101423540	−6.6718	963.2
7	Floralquinquenoside B	23652021	−6.5276	817.0

**Table 3:** ADME properties of the compounds.

Properties	Parameters	Floralquinquenoside C	Ginsenoside Rg6	Notoginsenoside T1	Floralquinquenoside B	Decision	Unit
Absorption	Water Solubility	−2.953	−3.43	−4.378	−2.988	Numeric	log mol/L
	CaCO-2 Permeability	−0.642	0.569	0.393	−0.63	Numeric	log Papp (10^−6^ cm/s)
	Intestinal Absorption (Human)	19.012	42.19	41.082	18.969	Numeric	% Absorbed
	Skin Permeability	−2.735	−2.735	−2.744	−2.735	Numeric	log Kp
	P-glycoprotein Substrate	Yes	Yes	Yes	Yes	Categorical	Yes/No
Distribution	Volume Distribution (VDss)	−0.556	−0.719	−0.749	−0.578	Numeric	log L/kg
	Fraction Unbound (Human)	0.422	0.312	0.308	0.42	Numeric	Fu
	BBB Permeability	−1.613	−1.111	−1.157	−1.683	Numeric	log BB
Metabolism	CYP1A2 Inhibitor	No	No	No	No	Categorical	Yes/No
	CYP2C19	No	No	No	No	Categorical	Yes/No
	CYP2C9	No	No	No	No	Categorical	Yes/No
	CYP2D6	No	No	No	No	Categorical	Yes/No
	CYP3A4	No	No	No	No	Categorical	Yes/No
Excretion	Total Clearance	0.458	0.485	0.334	0.593	Numeric	log mL/min/kg
	Renal OCT2 Substrate	No	No	No	No	Categorical	Yes/No

**Table 4:** Toxicity profile of ginseng compounds.

Ligands	Hepatotoxicity	Carcinogenicity	Immunotoxicity	Mutagenicity	Cytotoxicity
Floralquinquenoside C	Inactive	Inactive	Inactive	Inactive	Inactive
Ginsenoside Rg6	Inactive	Inactive	Inactive	Inactive	Inactive
Notoginsenoside T1	Inactive	Inactive	Inactive	Inactive	Inactive
Floralquinquenoside B	Inactive	Inactive	Inactive	Inactive	Inactive

## Data Availability

Data are contained within the article and Supplementary Materials.

## Supplementary Materials

The following supporting information can be downloaded at: https://www.mdpi.com/article/10.3390/ph18050648/s1, Table S1. List of Ginseng Compounds. Table S2. List of Target Proteins. Table S3. Information on the top 5 Enriched Go terms. Table S4. Detailed analysis of the top 10 enriched KEGG pathways. Table S5. Docking score of ginseng compound. Table S6. Drug-Likeness score of the compounds.

## Abbreviations

The following abbreviations are used in this manuscript:

HBP	High blood pressure
ACE	Angiotensin-converting enzyme
CA-I	Carbonic anhydrase-I
GO	Gene ontology
KEGG	Kyoto encyclopedia of genes and genomes
FDA	Food and drug administration
DL	Drug-likeness
PPI	Protein-protein interaction
STRING	Search tool for retrieval of interacting genes/proteins
DC	Degree of connectivity
CC	Closeness centrality
BP	Biological processes
MF	Molecular function
DAVID	Database for annotation, visualization, and integrated discovery
RCSB	Research collaboratory for structural bioinformatics
PDB	Protein data bank
RMSD	Root mean square deviation
RMSF	Root mean square fluctuation
MAPK	Mitogen-activated protein kinase
GPCR	G protein-coupled receptor
