# Supplementary material for: Exploring Ginseng Bioactive Compound’s Role in Hypertension Remedy: An In Silico Approach
Source: Pharmaceuticals (Basel). 2025 Apr 28;18(5):648. doi: 10.3390/ph18050648 (PMC12114325; doi:10.3390/ph18050648)
Supplement: Supplementary file 1 [file pharmaceuticals-18-00648-s001.zip › pharmaceuticals-3565930-supplementary.pdf]

Exploring Ginseng Bioactive Compound′s Role in Hypertension Remedy: An In Silico Approach

Sagar Kurmi [1,†], Rita Majhi [1,†], Hilal Tayara [2,*] and Kil To Chong [1,3,*]

[1] Department of Electronics and Information Engineering, Jeonbuk National University, Jeonju-si 54896, Jeollabuk-do, Republic of Korea

[2] School of International Engineering and Science, Jeonbuk National University, Jeonju-si 54896, Jeollabuk-do, Republic of Korea

[3] Advanced Electronics and Information Research Center, Jeonbuk National University, Jeonju-si 54896, Jeollabuk-do, Republic of Korea

* Correspondence: hilaltayara@jbnu.ac.kr (H.T.); kitchong@jbnu.ac.kr (K.T.C.)

† These authors contributed equally to this work.

Table S1

List of Ginseng Compounds

S.no	Compound Name	PubChem CID	S.no	Compound Name	PubChem CID
1	25(R)-Hydroxyprotopanaxadiol	158501	36	Floralginsenoside O	101423542
2	Floralginsenoside A	16655581	37	Floralginsenoside Ta	46224641
3	Floralginsenoside B	101423532	38	Floralginsenoside Tb	46224642
4	Floralginsenoside C	16655212	39	Floralginsenoside Tc	46224643
5	Floralginsenoside D	16655213	40	Floralquinquenoside A	23652020
6	Floralginsenoside E	101423533	41	Floralquinquenoside B	23652021
7	Floralginsenoside F	101423534	42	Floralquinquenoside C	23652173
8	Floralginsenoside G	101423535	43	Floralquinquenoside D	23652174
9	Floralginsenoside H	101423536	44	Floralquinquenoside E	23652175
10	Floralginsenoside I	16655580	45	Floranotoginsenoside A	163183858
11	Floralginsenoside J	101423537	46	Floranotoginsenoside B	163183855
12	Floralginsenoside K	101423538	47	Floranotoginsenoside C	163183856
13	Floralginsenoside Lb	102512867	48	Floranotoginsenoside D	163183857
14	Floralginsenoside M	101423540	49	Ginsenoside I	131751696
15	Floralginsenoside N	101423541	50	Ginsenoside II	101717751
16	Ginsenoside Km	102294900	51	Notoginsenoside N	101717750
17	Ginsenoside Rg6	91895489	52	Notoginsenoside O	21674162
18	Ginsenoside Rh5	10699455	53	Notoginsenoside P	21674163
19	Ginsenoside Ki	10699455	54	Notoginsenoside Q	21674164
20	Ginsenoside Rh6	131752646	55	Notoginsenoside R10	131752529
21	Ginsenoside Rh7	101096472	56	Notoginsenoside Rw1	163183850
22	Ginsenoside Rh8	85245726	57	Notoginsenoside S	21674165
23	Ginsenoside Rk1	11499198	58	Notoginsenoside T	21674166
24	Ginsenoside Rk2	90472238	59	Notoginsenoside T1	131752527
25	Ginsenoside Rk3	75412555	60	Notoginsenoside T2	131752528
26	Ginsenoside Rs5	102021585	61	Notoginsenoside T5	137796498
27	Ginsenoside R1	4483635	62	Notopanaxoside A	102383753
28	Isodehydroprotopanaxatriol	101855745	63	Panaxadione	25233029
29	Isoginsenoside Rh3	122196267	64	Quinquenoside L1	131751287
30	Notoginsenoside FP2	101838198	65	Quinquenoside L2	131751593
31	Notoginsenoside FT1	91973814	66	Quinquenoside L10	156599197
32	Notoginsenoside L	101130267	67	Yesanchinoside F	11018665
33	Notoginsenoside M	101130268	68	Yesanchinoside E	11136943
34	Yesanchinoside F	11018665	69	Yesanchinoside I	101201459
35	Yesanchinoside G	101201457	70	Yesanchinoside J	101201460

Table S2

List of Target Proteins

S.no	Target Protein	Gene Symbol	PDB ID
1	Type-1 angiotensin II receptor	AGTR1	P30556
2	Angiotensin-converting enzyme (ACE)	ACE	P12821
3	Peroxisome proliferator-activated receptor gamma	PPARG	P37231
4	Voltage-dependent L-type calcium channel subunit 1C	CACNA1C	Q13936
5	Voltage-dependent T-type calcium channel subunit 1G	CACNA1G	O43497
6	Voltage-dependent calcium channel subunit alpha-2/delta-1	CACNA2D1	P54289
7	Voltage-dependent L-type calcium channel subunit beta-2	CACNB2	Q08289
8	Voltage-dependent L-type calcium channel subunit alpha-1D	CACNA1D	Q01668
9	Voltage-dependent T-type calcium channel subunit alpha-1I	CACNA1I	Q9P0X4
10	Voltage-dependent L-type calcium channel subunit alpha-1S	CACNA1S	Q13698
11	Solute carrier family 12-member 2	SLC12A2	P55011
12	Solute carrier family 12-member 1	SLC12A1	Q13621
13	Solute carrier family 12-member 3	SLC12A3	P55017
14	Calcium-activated potassium channel subunit alpha-1	KCNMA1	Q12791
15	Carbonic anhydrase 1	CA1	P00915
16	Carbonic anhydrase 2	CA2	P00918
17	Interferon alpha/beta receptor 2	IFNAR2	P48551
18	Neuronal acetylcholine receptor subunit alpha-3	CHRNA3	P32297
19	Neuronal acetylcholine receptor subunit beta-4	CHRNB4	P30926
20	Neuronal acetylcholine receptor subunit alpha-10	CHRNA10	Q9GZZ6
21	Amiloride-sensitive sodium channel subunit alpha	SCNN1A	P37088
22	Amiloride-sensitive sodium channel subunit beta	SCNN1B	P51168
23	Amiloride-sensitive sodium channel subunit gamma	SCNN1G	P51170
24	Amiloride-sensitive sodium channel subunit delta	SCNN1D	P51172
25	Beta-1 adrenergic receptor	ADRB1	P08588
26	Beta-2 adrenergic receptor	ADRB2	P07550
27	Alpha-1A adrenergic receptors	ADRA1A	P35348
28	Alpha-2A adrenergic receptor	ADRA2A	P08913
29	Aromatic-L-amino-acid decarboxylase	DDC	P20711
30	Alpha-1B adrenergic receptor	ADRA1B	P35368
31	Alpha-1A adrenergic receptor	ADRA1A	P35348
32	ATP-sensitive inward rectifier potassium channel 1	KCNJ1	P48048

Table S3

Information on the top 5 Enriched Go terms

Go term	Go term	Gene count	Gene ratio (%)	p-Value	FDR
BP	adenylate cyclase-activating adrenergic receptor signaling pathway	5	50	2.97E-10	7.51E-08
BP	positive regulation of MAPK cascade	5	50	9.68E-07	6.13E-05
BP	G protein-coupled receptor signaling pathway	5	50	5.38E-04	0.022692
BP	regulation of vasoconstriction	4	40	6.33E-08	8.01E-06
BP	positive regulation of heart rate by epinephrine-norepinephrine	3	30	5.55E-07	4.68E-05
CC	plasma membrane	8	80	0.00208	0.077157
CC	receptor complex	3	30	0.003957	0.077157
CC	neuronal dense core vesicle	2	20	0.009014	0.117183
CC	cytoplasm	7	70	0.016444	0.160327
CC	caveola	2	20	0.031862	0.248527
MF	protein heterodimerization activity	5	50	1.69E-05	0.001299
MF	zinc ion binding	4	40	0.006783	0.07461
MF	Bradykinin receptor binding	2	20	9.32E-04	0.023928
MF	cyanamide hydratase activity	2	20	9.32E-04	0.023928
MF	norepinephrine binding	2	20	0.001864	0.028702

*GO, Gene Ontology; FDR value, False discovery rate < 0.05 p-value, Probability < 0.05; BP, Biological Process; CC, Cellular Component; MF, Molecular Function

Table S4

Detailed analysis of the top 10 enriched KEGG pathways

Term	Count	Gene ratio (%)	p-Value	FDR
GMP-PKG signaling pathway	6	60	2.61E-07	9.39E-06
Neuroactive ligand-receptor interaction	6	60	1.34E-05	1.60E-04
Adrenergic signaling in cardiomyocytes	5	50	1.04E-05	1.60E-04
Calcium signaling pathway	5	50	7.49E-05	5.39E-04
Renin secretion	4	40	3.70E-05	3.33E-04
Salivary secretion	4	40	1.03E-04	6.15E-04
Vascular smooth muscle contraction	3	30	0.007658	0.039382
Nitrogen metabolism	2	20	0.017183	0.077323
Renin-angiotensin system	2	20	0.023184	0.092738
Regulation of lipolysis in adipocytes	2	20	0.058515	0.210653

*KEGG, Kyto encyclopedia of genes and genomes; FDR, False discovery rate<0.05;

p-value, Probability value < 0.05.

Table S5

Docking score of ginseng compound

S.no	Compound Name	CID	Docking Score (kcal/mol)	Molecular formula	Molecular weight
1	Notoginsenoside O	21674162	-6.28668	$C_{52}H_{88}O_{21}$	1049.2g/mol
2	Ginsenoside Rk2	90472238	-6.8467	$C_{36}H_{60}O_{7}$	604.9g/mol
3	Ginsenoside Rk3	75412555	-6.41092	$C_{36}H_{60}O_{8}$	620.9g/mol
4	Ginsenoside Rh7	101096472	-6.09711	$C_{36}H_{60}O_{9}$	636.9g/mol
5	Floralginsenoside I	16655580	-8.40576	$C_{42}H_{72}O_{16}$	833g/mol
6	Quinquenoside L2	131751593	-7.66118	$C_{48}H_{82}O_{19}$	963.2g/mol
7	Floralginsenoside M	101423540	-6.67158	$C_{48}H_{82}O_{19}$	963.2g/mol
8	Notoginsenoside M	101130268	-7.79524	$C_{48}H_{82}O_{19}$	963.2g/mol
9	Notoginsenoside N	101717750	-7.03447	$C_{48}H_{82}O_{19}$	963.2g/mol
10	Floranotoginsenoside C	163183856	-6.92884	$C_{53}H_{90}O_{24}$	1111.3g/mol
11	Floranotoginsenoside D	163183857	-6.22497	$C_{53}H_{90}O_{23}$	1095.3g/mol
12	Floralginsenoside P	101423543	-6.9283	$C_{53}H_{90}O_{23}$	1095.3g/mol
13	Floralginsenoside k	101423538	-6.43001	$C_{50}H_{84}O_{21}$	1021.2g/mol
14	Quinquenoside L10	156599197	-6.84269	$C_{47}H_{80}O_{17}$	917.1g/mol
15	Quinquenoside L1	131751287	-6.39027	$C_{48}H_{80}O_{18}$	945.1g/mol
16	Floralginsenoside A	16655581	-6.31782	$C_{42}H_{72}O_{16}$	833g/mol

Table S6

Drug-Likeness score of the compounds

Compounds	Drug-Likeness	Compounds	Drug-Likeness
25(R)-Hydroxyprotopanaxadiol	0.48	Ginsenoside Km	0.45
Floralginsenoside B	0.44	Notoginsenoside T1	0.38
Floralginsenoside C	0.35	Ginsenoside Rh8	0.71
Floralginsenoside D	0.22	Ginsenoside Ki	0.45
Floralginsenoside F	0.26	Notoginsenoside T5	0.34
Floralginsenoside G	0.21	Floralginsenoside M	0.23
Floralginsenoside Lb	0.23	Floralquinquenoside B	0.28
Floralginsenoside N	0.31	Ginsenoside Rk2	0.39
Ginsenoside Rh5	0.47	Ginsenoside Rk3	0.39
Ginsenoside Rk1	0.41	Ginsenoside II	0.37
Ginsenoside Rs5	0.57	Floralginsenoside A	0.21
Isoginsenoside Rh3	0.4	Quinquenoside L2	0.41
Notoginsenoside FP2	0.24	Notoginsenoside N	0.36
Notoginsenoside FT1	0.45	Notoginsenoside M	0.28
Notoginsenoside L	0.26	Quinquenoside L10	0.24
Yesanchinoside G	0.38	Notoginsenoside O	0.27
Floralginsenoside O	0.29	Floralquinquenoside C	0.36
Floralginsenoside P	0.24	Ginsenoside Rg6	0.45
Floralginsenoside Tb	0.28	Floralginsenoside K	0.34
Floralquinquenoside A	0.31	Ginsenoside Rh7	0.48
Floralquinquenoside E	0.29	Ginsenoside R1	0.32
Floranotoginsenoside A	0.2	Yesanchinoside J	0.46
Notoginsenoside P	0.26		
Notoginsenoside Q	0.26		
Notoginsenoside R10	0.48		
Notoginsenoside Rw1	0.34		
Notoginsenoside S	0.24		
Notoginsenoside T	0.27		
Notoginsenoside T2	0.41		
Panaxadione	0.57		
Yesanchinoside F	0.52		
Yesanchinoside E	0.33		
